# On-Demand Therapy with Proton Pump Inhibitors for Maintenance Treatment of Nonerosive Reflux Disease or Mild Erosive Esophagitis: A Systematic Review and Meta-Analysis

**DOI:** 10.1155/2018/6417526

**Published:** 2018-08-12

**Authors:** Zubair Khan, Yaseen Alastal, Muhammad Ali Khan, Mohammad Saud Khan, Basmah Khalil, Shreesh Shrestha, Faisal Kamal, Ali Nawras, Colin W. Howden

**Affiliations:** ^1^Division of Gastroenterology and Hepatology, University of Toledo Medical Center, Toledo, OH, USA; ^2^Division of Gastroenterology and Hepatology, The University of Tennessee Health Science Center, Memphis, TN, USA

## Abstract

**Background:**

Proton pump inhibitors (PPIs) are widely used for the long-term management of gastroesophageal reflux disease (GERD). However, concerns about the cost and/or inconvenience of continuous maintenance PPI treatment have led to the evaluation of various alternative approaches.

**Aim:**

To assess the effectiveness of on-demand PPI therapy in the maintenance treatment of nonerosive reflux disease (NERD) or mild erosive esophagitis (EE).

**Methods:**

We searched MEDLINE, EMBASE, Web of Science, and Cochrane Library from inception until October 2, 2017, for randomized controlled trials (RCTs) comparing on-demand PPI versus placebo or daily PPI in the management of NERD or mild EE (Savary-Miller grade 1). Discontinuation of therapy during the trial was used as a surrogate for patient dissatisfaction and failure of symptomatic control. We calculated pooled odds ratios (OR) to evaluate the efficacy of on-demand PPI treatment. Separate analyses were conducted for studies comparing on-demand PPI with daily PPI and with placebo. Subgroup analysis was done based on NERD studies alone and on studies of both NERD and mild EE. These were analyzed using a random effects model.

**Results:**

We included 10 RCTs with 4574 patients. On-demand PPI was superior to daily PPI (pooled OR = 0.50; 95% confidence interval (CI) = 0.35, 0.72). On subgroup analysis in NERD patients only, pooled OR was 0.44 (0.29, 0.66). In studies including patients with NERD and mild EE, pooled OR was 0.76 (0.36, 1.60). For studies comparing on-demand PPI with placebo, pooled OR was 0.21 (0.15, 0.29); subgroup analyses of studies evaluating NERD only and studies conducted in NERD and mild EE showed similar results (pooled OR was 0.22 (0.13, 0.36) and 0.18 (0.11, 0.31), resp.).

**Conclusions:**

On-demand PPI treatment is effective for many patients with NERD or mild EE. Although not FDA-approved, it may be adequate for those patients whose symptoms are controlled to their satisfaction.

## 1. Introduction

Gastroesophageal reflux disease (GERD) is a common disorder of the upper gastrointestinal tract. The prevalence of reflux symptoms is steadily rising throughout the industrialized world [1]. An estimated 20–40% of Western adult populations report chronic heartburn or regurgitation symptoms [2]. Different manifestations of GERD include nonerosive reflux disease (NERD) and erosive esophagitis (EE). Complications of GERD, which are generally confined to EE patients, include ulceration, stricture, and Barrett's esophagus with attendant risk of esophageal adenocarcinoma [3]. NERD, the most frequent manifestation of GERD, is present in around 70% of patients and characterized by the presence of typical GERD symptoms associated with pathological acid reflux but the absence of demonstrable esophageal mucosal injury on endoscopy [4, 5]. Despite the absence of mucosal injury on endoscopy, many patients with NERD experience severe symptoms and impairment in quality of life that may be equivalent to, or greater than, seen in patients with EE [6, 7]. Acid-suppressive therapy with proton pump inhibitors (PPI) has proved to be the most effective treatment strategy for both NERD and EE [8–10]. PPIs have shown superiority over histamine H_2_-receptor antagonists for controlling symptoms as well as for healing erosions and preventing relapse [8, 10]. However, up to 75% patients with NERD and up to 90% of patients with EE may experience symptomatic relapse within six months of stopping treatment [11, 12]. Therefore, many patients subsequently receive long-term treatment to maintain adequate symptom control and, for EE patients, healing of erosions. However, this may have led to unnecessary use of these drugs, among NERD patients especially, adding to overall costs [13]. In the United States, the total expenditure for PPI treatment may be over $11 billion annually [14]. Due to the costs of PPI treatment, there have been efforts to develop effective and cost-efficient alternative long-term maintenance strategies for some GERD patients [15, 16], including “on-demand” PPI therapy, with patients taking a daily dose of a PPI when symptoms recur and stopping treatment when symptoms resolve. This is in contrast to intermittent treatment, in which patients take a regular daily dose of a PPI upon symptom relapse and continue it for a prespecified duration (typically 1 or 2 weeks) regardless of symptom response.

To evaluate the effectiveness of on-demand PPI treatment in patients with NERD or mild EE, we conducted a systematic review of randomized controlled trials (RCTs) comparing it with regular daily PPI treatment or placebo.

## 2. Methods

### 2.1. Data Sources and Search Strategy

We carried out this systematic review and meta-analysis in accordance with the guidelines of preferred reporting items for systematic review and meta-analysis (PRISMA) [17]. The search strategies were developed in Ovid MEDLINE, and the same keywords and subject headings were applied to Ovid EMBASE, Cochrane, Scopus, and ISI Web of Science databases from inception through November 2, 2016. The search terms included “Esophagitis” OR “Gastroesophageal reflux” OR “GERD” OR “Nonerosive reflux disease” OR “NERD” OR “Erosive esophagitis” OR “EE” AND “Proton pump inhibitors” OR “PPIs” AND “on-demand” OR “on demand” OR “daily” AND “Placebo.” A medical librarian with more than 20 years of experience performed this search.

### 2.2. Study Selection and Inclusion and Exclusion Criteria

Two authors (Z.K. and Y.A.) searched for original studies based on the previously defined search strategy. We searched for RCTs comparing on-demand PPI treatment with either placebo or daily PPI in the maintenance treatment of NERD and/or mild EE. NERD was defined as the presence of classic GERD symptoms in the absence of esophageal mucosal injury during upper endoscopy (Savary-Miller Grade 0 and LA class M). Mild EE was defined as having esophagitis with Savary-Miller Grade 1 or LA class A. The main outcome measure used to assess treatment efficacy was discontinuation of therapy during the trial. Continuation of therapy during a trial was used as a surrogate for patient satisfaction and control of GERD symptoms. Hence, the proportion of patients who discontinued therapy during a trial was taken as an indirect measure of failure of symptomatic control of GERD. Studies were excluded if they did not contain raw or usable data or were published only in abstract form. We also excluded duplicate publications, expert opinion, and letters. We also searched bibliographies of retrieved articles to enhance the yield of our search strategy. All articles were downloaded into Endnote 7.0, a bibliographic database manager, and any duplicate citations were identified and removed.

### 2.3. Data Extraction and Quality Assessment

Two reviewers (Z.K. and Y.A.) assessed the eligibility of selected studies and extracted data using customized data extraction forms. Any disagreement between reviewers was discussed with a third reviewer (M.A.K.), and agreement was reached by consensus. Extracted data included study design, the year and country of publication, inclusion and exclusion criteria, PPI regimen used for on-demand and continuous groups, classification of esophagitis, outcome studied (number of patients discontinuing on-demand treatment due to inadequate symptom control), follow-up duration, and patient demographics.

We used the Cochrane tool for assessing risk of bias for RCTs. Two reviewers (Z.K. and Y.A.) performed quality assessment with any disagreement to be discussed with a third reviewer (M.A.K.). We used the GRADE framework to interpret our findings [18].

### 2.4. Data Synthesis and Statistical Analysis

Our main outcome of interest was the effectiveness of on-demand PPI treatment versus placebo or daily PPI in the management of NERD and/or mild EE. The primary efficacy endpoint used was the premature discontinuation of treatment. We analyzed pooled data using a random effects model and calculated odds ratios (ORs) with their 95% confidence interval (CI). We conducted separate analyses for studies comparing on-demand PPI with daily PPI and with placebo. We performed additional subgroup analyses based on NERD studies alone and on studies including patients with either NERD or mild EE. Cochrane's *Q* test and *I* ^2^ statistics were used to assess heterogeneity among studies. A *P* value of <0.1 for the Cochrane *Q* test or an *I* ^2^ value of >50% signified the presence of heterogeneity.

We constructed funnel plots and used Egger's precision test to assess publication bias. Statistical analysis was performed using RevMan, version 5.3 for Windows (Cochrane Collaboration, the Nordic Cochrane Center, Copenhagen, Denmark, 2014).

## 3. Results

### 3.1. Search Strategy Yield and Identification of Studies

The search strategy identified 409 articles, of which 35 were removed as duplicates. Of the remaining 374 articles, 347 were removed after title and abstract review. The remaining 27 full-text articles were reviewed, of which 10 RCTs [19–28] with 4574 patients were included in the meta-analysis as shown in PRISMA flowchart (Supplementary Materials (available here)). Among the patients, 2797 received on-demand PPI, 843 received daily PPI and 934 received placebo. Four RCTs [19–22] compared a daily PPI regimen with on-demand treatment. Three of these were confined to patients with NERD [19–21], while the fourth [22] also included patients with mild EE. Six trials compared on-demand treatment with placebo [23–28]; four only included patients with NERD [23–26], and two included both patients with NERD or mild EE [27, 28]. Tables 1 [Table tab2]–3 highlight the characteristics of included studies.

### 3.2. Meta-Analysis

#### 3.2.1. On Demand versus Daily PPI

**Figure 1:**
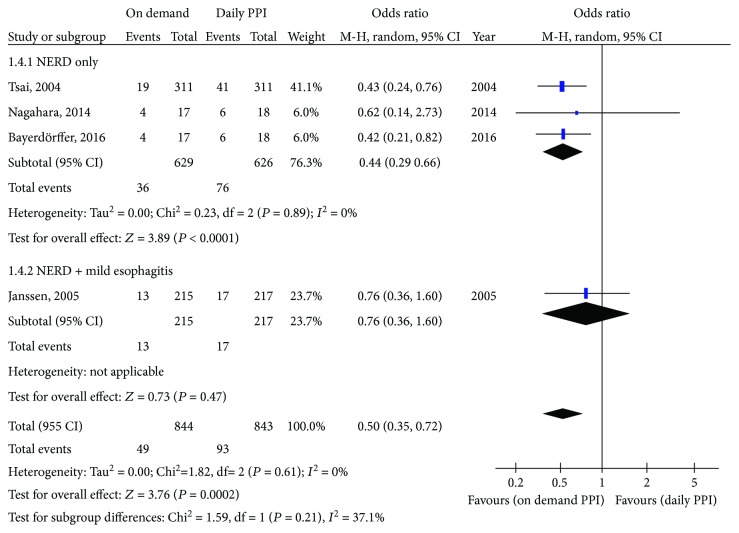
Forest plot comparing on-demand PPI with daily PPI.

For studies comparing on-demand PPI therapy with daily PPI therapy, 5.8% of patients discontinued treatment in the on-demand group compared to 11.0% in the daily PPI group. The pooled OR with 95% confidence interval (CI) was 0.50 (0.35, 0.72), with no heterogeneity (*I* ^2^ = 0%) (Figure 1). On subgroup analysis of the three RCTs that included only NERD patients, results were similar; 5.7% of patients in the on-demand PPI group and 12.1% of patients in the daily PPI group discontinued treatment prematurely (OR = 0.44; 95% CI = 0.29 to 0.66). However, on subgroup analysis of the study that included patients with either NERD or mild EE, there was no significant difference between treatments (OR = 0.76; 95% CI = 0.36 to 1.60). With on-demand treatment, 6.0% of patients discontinued treatment prematurely compared with 7.8% on daily PPI treatment.

#### 3.2.2. On-Demand PPI Treatment versus Placebo

**Figure 2:**
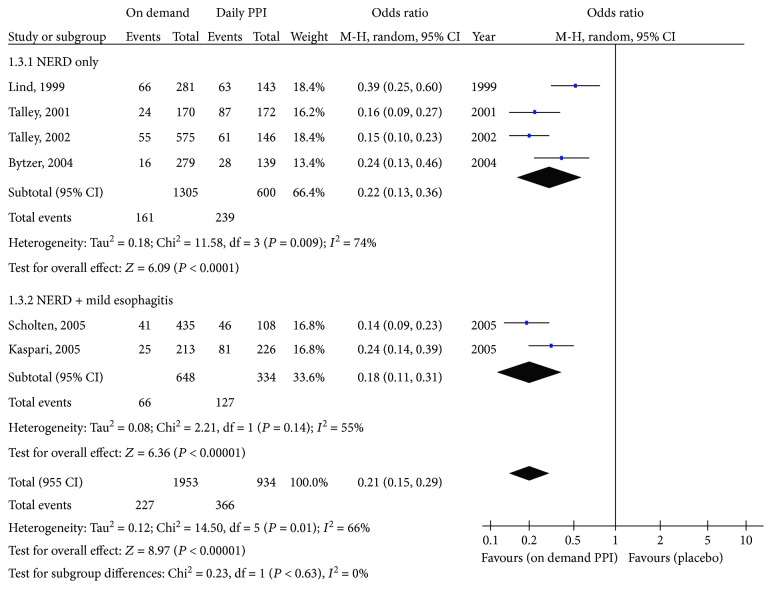
Forest plot comparing on-demand PPI with placebo.

The proportions of patients prematurely discontinuing treatment were 11.6% with on-demand PPI therapy and 39.2% with placebo (pooled OR = 0.21; 95% CI = 0.15 to 0.29). There was significant heterogeneity among studies (*I* ^2^ = 66%) (Figure 2). On subgroup analysis of studies conducted only in NERD patients, on-demand PPI treatment was superior to placebo; proportions of patients prematurely discontinuing treatment were 12.3% and 39.8%, respectively (OR = 0.22; 95% CI = 0.13 to 0.36). Among studies evaluating patients with either NERD or mild EE, proportions of patients discontinuing treatment prematurely were 10.2% and 38.0% (OR = 0.18; 95% CI = 0.11 to 0.31), respectively.

## 4. Discussion

We found that on-demand PPI therapy was superior to both placebo and daily PPI therapy as maintenance treatment for patients with NERD or mild EE. In general, more patients were willing to continue on-demand PPI treatment than either of the alternatives studied. Furthermore, adherence to treatment and patient satisfaction were higher with on-demand PPI treatment compared to continuous PPI treatment. On-demand treatment may help to improve overall patient satisfaction since patients may feel more in control of their treatment and can take a dose of PPI according to their perceived needs and symptoms [18]. However, the usefulness of on-demand PPI treatment compared to daily PPI was less obvious when patients with mild EE were included in the analysis.

Our analysis is different from two previously conducted analyses. Jiang et al. [29] concluded that on-demand treatment with PPIs is superior to continuous or placebo therapy, but did not perform subgroup analysis of NERD and mild EE. A recently published Cochrane review showed that on-demand deprescribing may lead to an increase in gastrointestinal symptoms (e.g., dyspepsia and regurgitation) in patients with NERD or mild grades of EE (Los Angeles grades A and B or Savary-Miller grades 1 and 2) [30]. Besides having a broad definition for on-demand deprescribing, this review did not differentiate NERD from mild EE; Savary-Miller grading for most of the included patients was >1. In our analysis, the only included were patients with Savary-Miller grade 0 (NERD) or 1 (mild EE). We excluded studies that included EE patients with Savary-Miller grade higher than 1, since those would represent moderate and severe EE.

Since the risk of progression of NERD or mild EE to more advanced disease is low, symptom control is the main objective of management [31–33]. Many patients experience intermittent symptoms of short duration. These symptoms can be effectively managed with on-demand PPI treatment. This symptom-driven approach for the long-term management of NERD also simulates many patients' actual use of these medicines [16]. Many patients who are prescribed daily PPI treatment may actually consume them on an as needed basis. Up to 29% of patients who were prescribed continuous PPI treatment decreased the frequency of use without a recommendation from their providers, and only 21% of patients prescribed continuous PPI treatment fill their prescriptions in a manner to remain fully compliant with the recommended dosing schedule [34, 35]. The most commonly cited reasons for not continuing treatment are inconvenience and cost. However, studies [36–38] evaluating the on-demand strategy versus continuous PPI treatment for EE have shown that the on-demand strategy is inferior. On-demand PPI treatment is, therefore, not appropriate or adequate for patients with EE of Savary-Miller grade 2 or above.

As well as patient preference and satisfaction for on-demand PPI treatment, cost of long-term treatment is also important. The consistently demonstrated efficacy and the favorable safety profile of PPI treatment have led to its widespread use in GERD patients [13]. There has been a substantial increase in the cost of GERD management with medicines contributing to overall costs. This has led to increasing concern from health care authorities and third-party payers [36]. On-demand PPI treatment might reduce overall consumption by up to one third. Tsai et al. [19], who compared on-demand esomeprazole with daily lansoprazole in 622 patients for six months, found that patients in the esomeprazole on-demand group took treatment on approximately one-third of the days (0.3 times per day) whereas those receiving lansoprazole daily took treatment on approximately 4 of every 5 days (0.8 times per day) despite being instructed to take them every day. Bayerdörffer et al. [21] found similar results with mean daily drug consumption of 0.41 tablets in the on-demand group and 0.91 tablets in the continuous daily group. Therefore, on-demand treatment may be a cost-effective strategy for the long-term management of patients with NERD or mild EE. It can be effective in controlling symptoms and is convenient for, and acceptable to, many patients. Those patients with NERD or mild EE who do not obtain adequate relief with on-demand PPI treatment can be considered for regular, daily PPI treatment in the lowest effective dose.

The studies included in our analysis defined NERD based on symptoms and endoscopic findings without physiological demonstration of abnormal gastroesophageal reflux (acidic or weekly acidic), which could be a limitation. Also, most of the studies used only Savary-Miller grading for classifying esophagitis. The studies that classified esophagitis using the LA classification were very limited and did not differentiate between LA grades A and B. We tried to be conservative while defining mild esophagitis and so confined our analysis to studies with either LA grades M or A and considered LA grade B to be indicative of moderate—rather than mild—esophagitis.

## 5. Conclusion

On-demand PPI treatment appears to be effective for the long-term management of many patients with NERD or mild EE. After the initial control of symptoms with a course of PPI treatment, on-demand PPI treatment can be appropriately considered for the long-term management of such patients.

## Figures and Tables

**Table 1:** Characteristics of studies comparing on-demand PPI with daily PPI.

References	Country	Center (multi, single)	Study design	Inclusion criteria	Exclusion criteria	PPI regimen for continuous therapy	PPI regimen for on-demand therapy	Outcome studied	Follow-up period	Esophagitis class
[19]	UK (92 general practices, 28 hospitals)	Multicenter	Single-blind (investigator), randomized, parallel group study	NERD with resolution of heartburn after 2 to 4 weeks of esomeprazole 20	Patients with persistent heartburn and structural diseases	Lansoprazole 15 mg PO once daily	Esomeprazole 20 mg	Time to discontinuation due to unwillingness to continue	6 months	Not applicable

[20]	Single university hospital Japan	Single center	Prospective parallel randomized open-label study	Patients with modified LA class M after having 8-week treatment with PPIs	Patients with cancer, serious liver disease, kidney disease, heart disease, a hematological disorder, gastric ulcers, and/or duodenal ulcers	Omeprazole 20 mg	Omeprazole 20 mg	Symptom relief at 4, 8, 16, and 24 weeks in each study group with relief from symptoms as the primary endpoint	6 months	Modified LA class M

[21]	Austria, France, Germany, South Africa, and Spain (61 centers)	Multicenter	Open-label, randomized, parallel group	NERD who were heartburn-free after 4-week treatment with esomeprazole 20 mg daily	Reflux esophagitis	Esomeprazole 20 mg	Esomeprazole 20 mg	Discontinuation due to unsatisfactory treatment	6 months	Not applicable

[22]	58 active centers: 29 in Germany, 12 in France, 11 in Switzerland, and 6 in Hungary	Multicenter	Open-label, randomized, parallel group	NERD + mild esophagitis treated with pantoprazole 20 mg PO daily for 4 weeks	Patients with persistent symptoms and heartburn, erosive esophagitis	Pantoprazole 20 mg	Pantoprazole 20 mg	The symptoms (as assessed in the patient's diary) were considered controlled until the time of failure, which was defined as the first point at which one of the following events occurred: (1) GERD symptoms of at least moderate severity were present for 3 or more consecutive days despite medication (event time = the first of these 3 days); (2) use of >1 tablet of study medication on >3 consecutive days (event time = the first of these 3 days); or (3) premature withdrawal from the study due to lack of efficacy (event time = the date of withdrawal)	6 months	Savary-Miller grade 0 or 1

**Table 2 tab2:** Characteristics of studies comparing on-demand PPI with placebo.

References	Country	Center (multi, single)	Study design	Inclusion criteria	Exclusion criteria	PPI regimen for continuous therapy	PPI regimen for on-demand therapy	Primary outcome studied	Follow-up period	Esophagitis class
[23]	Sweden & Denmark (25 centers)	Multicenter	Double-blind, randomized, placebo controlled	NERD with resolution of heartburn after short-term treatment (4 to 8 weeks)	Erosive, ulcerative PUD	N/A	Omeprazole 20, omeprazole 10, placebo	Discontinuation of medicine due to unwillingness to continue	6 months	N/A

[24]	65 centers in Denmark, Finland, Norway, and Sweden	Multicenter	Randomized, double-blind, parallel group	Endoscopy-negative GERD treated with 4 weeks of omep 20 or Eso 20	Patients requiring concomitant drugs, NSAIDS, quinidine, etc. excluded	N/A	Esomeprazole 20 mg on demand, placebo on demand	Time to discontinuation of on-demand therapy due to unwillingness to continue	6 months	N/A

[25]	116 centers in the UK, the Republic of Ireland, and Canada	Multicenter	Randomized, double-blind, parallel-group study	Patients with NERD treated with Eso 40, 20, or Omep 20 for 4 weeks	Patients requiring concomitant drugs, NSAIDS, quinidine, etc. excluded	N/A	Esomeprazole 40 mg on demand, esomeprazole 20 mg on demand, placebo on demand	Time to study discontinuation due to unwillingness to continue for any reason	6 months	N/A

[26]	International (Greece, Italy, the Netherlands, Spain, France, Portugal, Sweden, Denmark, Ireland, Belgium, United Kingdom, Russia, Poland, and Lithuania)	Multicenter	Randomized, double-blind, placebo-controlled, withdrawal study	NERD treated with rabeprazole 10 mg PO daily for 4 weeks	Patients with erosive disease and no relief of heartburn in acute 4-week phase	N/A	Rabeprazole 10 mg on demand, placebo on demand	The proportion of patients discontinuing treatment in the on-demand phase because of inadequate heartburn control	6 months	N/A

[27]	Germany 36 centers	Multicenter	Randomized, double-blind, placebo-controlled, parallel-group comparison	Nonerosive GERD or reflux esophagitis grade 1 according to Savary–Miller classification underwent 4 weeks of pantoprazole 20 mg PO OD for 4 weeks	Patients symptomtic after acute phase or nonerosive GERD or reflux esophagitis grade 2 to 4 according to Savary–Miller classification	N/A	Pantoprazole 40 mg on demand, pantoprazole 20 mg on demand, placebo on demand	Discontinuation rate due to insufficient control of heartburn	6 months	Savary-Miller grade 0 or 1
[28]	40 centers in Germany and five in Lithuania	Multicenter	Randomized, double-blind, placebo-controlled parallel-group comparison	Nonerosive GERD or reflux esophagitis grade 1 according to Savary–Miller classification underwent 4 weeks of pantoprazole 20 mg PO OD for 4 weeks	Patients symptomtic after acute phase or nonerosive GERD or reflux esophagitis grades 2 to 4 according to Savary–Miller classification	N/A	Pantoprazole 20 mg on demand, placebo on demand	The number of patients unwilling to continue the therapy and the corresponding reasons for it were analyzed using the Kaplan–Meier analysis	7 months	Savary-Miller grade 0 or 1

**Table 3:** Patient characteristics and demographics of included trials.

Study	*N*, on demand/cont	Mean age ± SD	Male (%)
Tsai et al. [19]	(1) Eso 20 mg on demand = 311	51 ± 13.8	46%
	(2) Lanso 15 mg continuous = 311	51 ± 13.8	41.8%

Nagahara et al. [20]	Omeprazole 20 mg continuous = 18	56.2 ± 12.8	21/35 = 60%
	Omeprazole 20 mg on demand = 17		

Bayerdörffer et al. [21]	(1) Eso 20 mg on demand = 301	48.2 ± 13.6	40.5%
	(2) Eso 20 mg continuous = 297	47.6 ± 15.1	43.8%

Janssen et al. [22]	Pantoprazole 20 mg on demand = 215	50.4 (SD 13.6)	46.5%
	Pantoprazole 20 mg continuous = 217	51.8 (SD 13.5)	47.5%

Lind et al. [23]	(1) Omeprazole 20 on demand = 139 (*n*)	52 (19–79) *R*	38.1%
	(2) Omeprazole 10 on demand = 142	51 (20–81) *R*	45.8%
	(3) Placebo = 143	48 (20–79) *R*	42.7%

Talley et al. [24]	(1) Eso 20 mg on demand = 170	49 (19–78) *R*	55%
	(2) Placebo on demand = 172	49 (21–79) *R*	57%

Talley et al. [25]	(1) Esomeprazole 40 mg on demand = 293	48	46.1%
	(2) Esomeprazole 20 mg on demand = 282	48.4	47.9%
	(3) Placebo on demand = 146	48.2	39.7%

Bytzer et al. [26]	(1) Rabeprazole 10 mg on demand = 279	47 (0.81 SE)	44%
	(2) Placebo on demand = 139	48 (1.23 SE)	41%

Scholten et al. [27]	Pantoprazole 40 mg on demand = 218	54 ± 14	47.3%
	Pantoprazole 20 mg on demand = 217	52 ± 14	52.5%
	Placebo on demand = 108	52 ± 14	53.7%

Kaspari et al. [28]	Pantoprazole 20 mg on demand = 213	50.7 ± 13.7 years	46%
	Placebo on demand = 226	51.0 ± 14.5 years	43.3%

## Abbreviations

PPIs: Proton pump inhibitors
GERD: Gastroesophageal reflux disease
EE: Erosive esophagitis
NERD: Nonerosive reflux disease
PRISMA: Preferred reporting items for systematic review and meta-analysis
RCT: Randomized clinical trial
OR: Odds ratio.

## Supplementary Materials

Supplementary Materials PRISMA flow chart for study selection.
