# Supplementary material for: On-Demand Therapy with Proton Pump Inhibitors for Maintenance Treatment of Nonerosive Reflux Disease or Mild Erosive Esophagitis: A Systematic Review and Meta-Analysis
Source: Gastroenterol Res Pract. 2018 Aug 12;2018:6417526. doi: 10.1155/2018/6417526 (PMC6109549; doi:10.1155/2018/6417526)
Supplement: Supplementary Materials — PRISMA flow chart for study selection. [file 6417526.f1.docx]

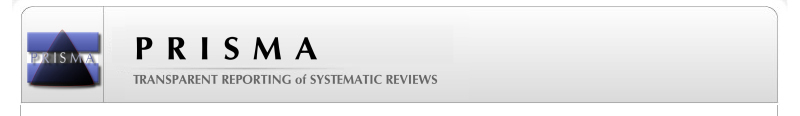
**PRISMA 2009 Flow Diagram**

Full-text articles excluded, with reasons
(n = 17 )

Review articles 6

Studies with no relevant data 8

Non-comparative studies 3

Records identified through databases searching:
n= 409

PubMed (n=20)

Embase (n=20)

Cochrane (n=301)

Web of Science (n=68)

Additional records identified through other sources
(n=0)

Records after duplicates removed
(n = 374)

Studies included in quantitative synthesis (meta-analysis)
(n = 10 )

Studies included in qualitative synthesis
(n = 10 )

Full-text articles assessed for eligibility
(n = 27 )

Records excluded
(n = 347 )

Records screened
(n = 374 )

## Identification

## Screening

## Eligibility

## Included
